# Imaging of MASLD: clinical standards and novel methods

**DOI:** 10.1007/s00125-026-06799-y

**Published:** 2026-07-16

**Authors:** Marc Jonuscheit, Vera B. Schrauwen-Hinderling

**Affiliations:** 1 Institute for Clinical Diabetology, German Diabetes Center, Leibniz Center for Diabetes Research at Heinrich Heine University Düsseldorf, Düsseldorf, Germany; 2 German Center for Diabetes Research (DZD), Partner Düsseldorf, München-Neuherberg, Germany

**Keywords:** Fibrosis, Imaging, Inflammation, Magnetic resonance elastography, Magnetic resonance spectroscopy, MASLD, Metabolic dysfunction-associated steatotic liver disease, MRI, Positron emission tomography, Review, Steatosis, Ultrasound

## Abstract

Non-invasive imaging is central to the diagnosis and staging of metabolic dysfunction-associated steatotic liver disease (MASLD). Various non-invasive means are used to reliably diagnose steatosis, whereas liver biopsy remains the gold standard for MASLD staging, as it enables assessment of the severity of fibrosis and inflammation. Importantly, imaging modalities have the potential to serve as non-invasive alternatives to biopsy in the future. This review summarises established and emerging imaging techniques for the diagnosis and staging of MASLD, focusing on their measurement principles, diagnostic performance and practical limitations. Conventional ultrasound remains the most widely used first-line modality for steatosis screening owing to its low cost and broad availability, but it lacks sensitivity for mild steatosis and is strongly operator and body habitus dependent. Quantitative ultrasound extensions such as the controlled attenuation parameter improve steatosis assessment, while transient elastography additionally measures liver stiffness, which is a surrogate marker for fibrosis. Computed tomography permits density-based assessment of steatosis and focal lesions but is limited by radiation exposure and poor sensitivity for low hepatic lipid (HL) content. MRI-derived proton density fat fraction and proton magnetic resonance spectroscopy are considered non-invasive reference standards for quantifying HL content, with high sensitivity and specificity even at low fat fractions, albeit at a higher cost and with more restricted availability. For fibrosis staging based on liver stiffness, magnetic resonance elastography currently offers the highest non-invasive accuracy across fibrosis stages and reduces sampling error by probing large liver volumes, whereas transient elastography is more widely implemented in routine care owing to its broader availability. Additional MRI-based approaches, including diffusion-weighted imaging and T_1_-based mapping techniques, provide complementary information on tissue microstructure, reflecting inflammatory and fibrotic processes, although their specificity and standardisation are still under evaluation. Nuclear imaging with positron emission tomography and single-photon emission computed tomography enables functional and molecular characterisation. Novel tracers targeting fibroblast activation protein, macrophage subsets or activated hepatic stellate cells, together with targeted MRI contrast agents and nanoparticle-based probes, hold promise for more specific imaging of fibrogenesis and inflammation. Beyond structural changes, multinuclear magnetic resonance spectroscopy and specialised positron emission tomography tracers allow in vivo assessment of hepatic energy metabolism, mitochondrial function, glycogen dynamics and substrate fluxes, which is particularly relevant for mechanistic studies, increasing our understanding of MASLD pathology, but are currently confined to research settings. Future developments will likely emphasise multiparametric and multimodal strategies integrating quantitative imaging biomarkers with clinical and biochemical data to refine risk stratification, enable sensitive treatment monitoring and support precision medicine approaches in MASLD.

**Graphical Abstract:**

**Figure:**
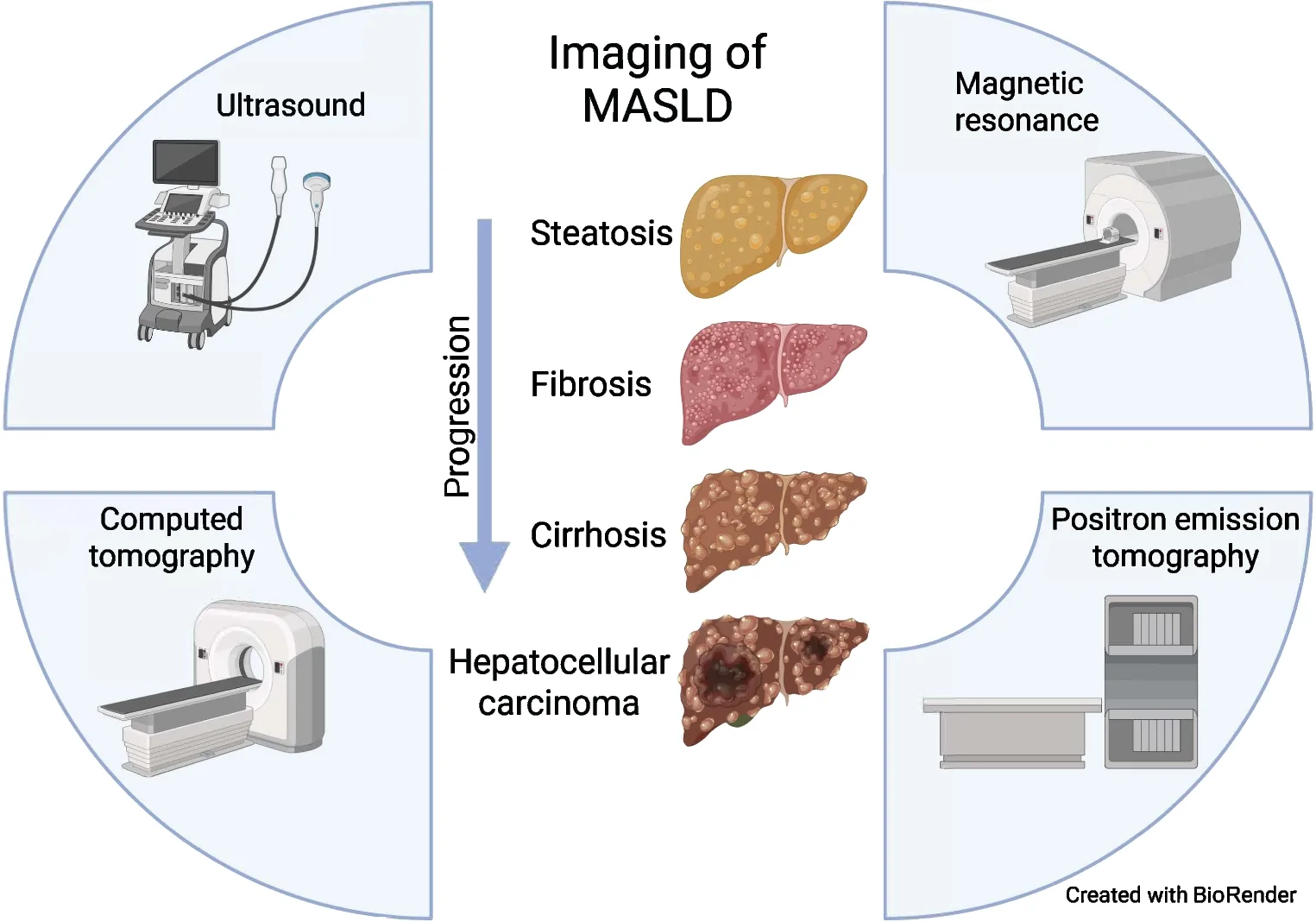

**Supplementary Information:**

The online version contains a slideset of the figures for download available at https://doi.org/10.1007/s00125-026-06799-y.

## Introduction to MASLD

Given the high prevalence of metabolic dysfunction-associated steatotic liver disease (MASLD) and the significant risk of irreversible disease progression to cirrhosis and hepatocellular carcinoma (HCC), early detection and accurate disease staging are of utmost importance. Among all of the histopathological features, fibrosis stage is the strongest predictor of liver-related morbidity and mortality in individuals with MASLD [1]. The current clinical practice guidelines of the European Association for the Study of the Liver (EASL), European Association for the Study of Diabetes (EASD) and the European Association for the Study of Obesity (EASO) on the management of MASLD recommend the use of non-invasive scores based on combinations of blood tests or combinations of blood tests with imaging techniques for the detection of fibrosis, as their diagnostic accuracy is higher than that of standard liver enzyme testing [2]. Moreover, a multistep approach consisting of a blood-based score, such as the Fibrosis-4 (FIB-4) index, and an established imaging technique are recommended for staging when fibrosis is still suspected or in high-risk groups such as those with type 2 diabetes. These recommendations highlight the evolving role of non-invasive imaging biomarkers as part of a graduated diagnostic strategy.

MASLD is more prevalent and more severe in people living with type 2 diabetes than in individuals without diabetes [3]. A large multicentre study in individuals with biopsy-proven MASLD demonstrated significantly faster fibrosis progression in people with type 2 diabetes than in those without, with cumulative incidences of progression by one or more stage of 24% vs 20% at 4 years, 60% vs 50% at 8 years, and 93% vs 76% at 12 years [4]. Another study found that, among individuals with type 2 diabetes, even when a low threshold of alanine aminotransferase (ALT) was used, 38% had advanced fibrosis (F3 or F4) and 58% had liver inflammation; moreover the FIB-4 index showed moderate standalone performance for advanced fibrosis and metabolic dysfunction-associated steatohepatitis (MASH), respectively [5]. This has important implications for screening, risk stratification and the interpretation of non-invasive tests in clinical practice and underscores that novel imaging tools should be validated carefully in people with type 2 diabetes to assess potential bias in this higher risk group.

Liver biopsy remains the gold standard for the detection of MASH. Therefore, biopsy plays a pivotal role in establishing a definitive diagnosis, despite its clear limitations, including the risk for complications, high cost and resource use, and the small volume of liver tissue sampled [6]. Consequently, there is a growing need for non-invasive modalities, which are emerging as valuable tools for assessing biomarkers not only for the diagnosis of MASLD/MASH, but also for longitudinal monitoring and evaluation of therapeutic responses, thereby enabling a more comprehensive understanding of the disease. In this context, metabolic changes at the cellular level that drive the progression of MASLD can often be detected much earlier using imaging methods. For example, when examining energy metabolism, changes in redox state and mitochondrial capacity can be observed during obesity, before MASLD/MASH develops [7, 8]. The aim of this review is to highlight the non-invasive imaging modalities and techniques available for the detection and investigation of MASLD.

## Imaging modalities for in vivo liver evaluation

Non‑invasive imaging plays an important role in the assessment of hepatic steatosis, inflammation and fibrosis in MASLD. Each modality offers unique advantages regarding accessibility, quantification capability and biological specificity (Table 1). The following overview summarises the most relevant imaging techniques currently used for in vivo liver evaluation in MASLD, outlining their measurement principles, technical requirements, spatial resolution, contrast mechanisms, availability, costs and limitations.

**Table 1:** Summary of non-invasive imaging modalities for MASLD detection

Imaging modality	Basic principle	Spatial resolution	Contrast mechanism in MASLD	Availability	Costs	Limitations in MASLD	Safety
US	Acoustic waves	+	Echogenicity (steatosis >20%)	+++	+	Low sensitivity for the detection of mild steatosis Reduced accuracy in individuals with obesity	No radiation
TE	Shear wave propagation	+	Stiffness for fibrosis, CAP for steatosis	+++	+	Reduced accuracy in individuals with obesity (20% failure rate) Requires specific operator training	No radiation
CT	X-ray attenuation	+++	Density for steatosis, contrast for lesions	+++	+	Low sensitivity for the detection of mild steatosis Iron is a confounder	Ionising radiation
MRI	NMR of ^1^H in a strong magnetic field	+++	PDFF for steatosis, DWI-MRI and T_1_/T_1_ρ mapping for fibrosis	++	++	Prone to motion artefacts Weight restrictions	No radiation; contraindications include magnetic implants and claustrophobia
MRE	2D/3D shear waves + phase-contrast MRI	++	Stiffness maps for fibrosis	+	++	As for MRI limitations + need for breath-hold	As for MRI
MRS	Frequency-specific metabolite signals	+	^1^H for steatosis, ^31^P/^13^C for metabolites	+	++	Prone to motion artefacts Requires longer examination times SNR limited in those with high BMI when using surface coils	As for MRI
PET/SPECT	γ-photons from radiotracers	++	Tracer uptake for fibrosis, inflammation and metabolism	++	+++	No routine MASLD tracers High cost	Ionising radiation; radiotracer infusion and exposure
Hybrid imaging systems (PET/CT, PET/MR, SPECT/CT)	Functional + anatomical co-registration	+++	Multiparametric (structural and molecular)	+	+++	High cost Limited clinical availability	Ionising radiation; radiotracer infusion and exposure

PDFF, proton density fat fraction; SNR, signal-to-noise ratio

### Ultrasound

**Fig. 1:**
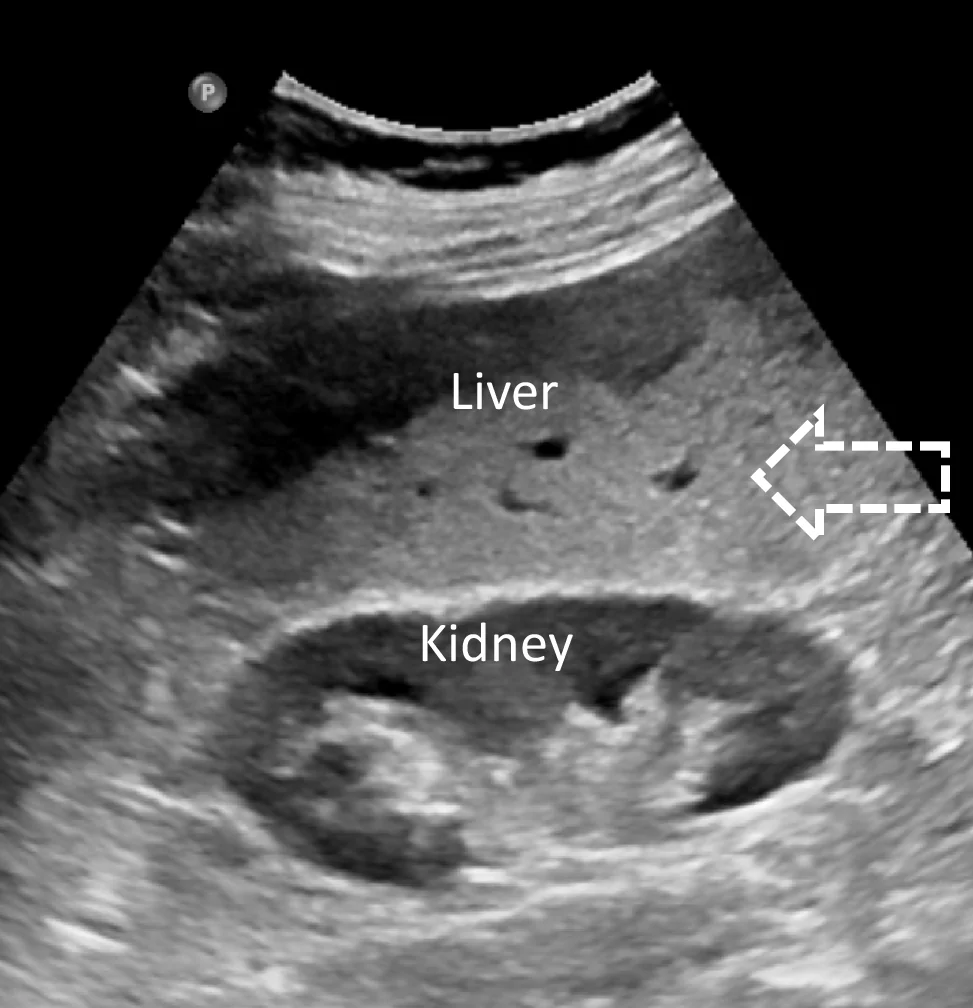
US image of the abdomen for the detection of hepatic steatosis. The image shows areas of the liver with severe steatosis (dotted arrow), recognisable by increased echogenicity compared with the renal cortex. At the top of the image, a darker (less echogenic) area of liver is visible, representing healthy (non-steatotic) tissue. Image courtesy of T. van de Weijer (Department of Radiology, Maastricht University Medical Center, Maastricht UMC+, the Netherlands). This figure is available as part of a downloadable slideset

**Fig. 2:**
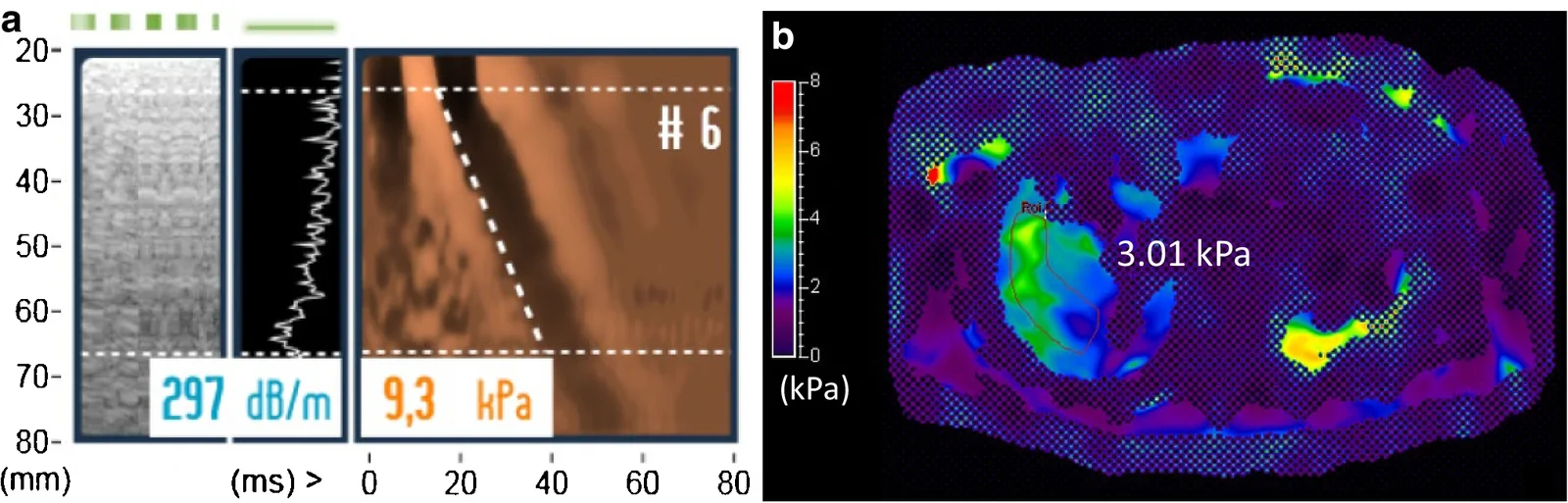
Examples of (**a**) CAP and liver stiffness estimated by US TE and (**b**) liver stiffness assessed by MRE in individuals with fibrosis. (**a**) The image on the left illustrates the time mode signal and shows low tissue heterogeneity and centred probe placement, ensuring that the signal is artefact-free, while the central image represents the amplitude mode signal, reflecting acoustic properties (attenuation of the signal) along the measurement line. The image on the right displays the shear wave propagation (elastography), which is used to quantify liver stiffness. A parallel margin shear wave (diagonal dotted line) indicates that the shear wave speed is correctly estimated and no rib echo interference is present. (**b**) Commercial vendor software automatically processes colour-coded MRE stiffness maps. The grid shows the areas that the algorithm identifies as unreliable. For the assessment of liver stiffness, regions are manually identified, from which the mean stiffness value is derived. Note that both US TE and MRE have different thresholds for the presence of fibrosis and their numerical values should not be interpreted interchangeably, as each method requires technique-specific cut-offs for fibrosis staging. Fig. 2a courtesy of F. Battiato, D. M. Méndez Cárdenas and M. Varadinova (Clinical Research Center at the Institute for Clinical Diabetology, German Diabetes Center). This figure is available as part of a downloadable slideset

Ultrasound (US) is based on the emission and detection of high‑frequency acoustic waves. In abdominal applications, its spatial resolution depends on the transducer frequency (1–5 MHz) and the volunteer body habitus, whereas the image contrast is determined by differences in acoustic impedance between tissues. Lipid-rich tissue (e.g. liver tissue with a high density of lipid vacuoles) is echogenic and therefore appears brighter than lean liver. For routine assessment of hepatic lipid (HL) content, hepatic echogenicity relative to surrounding structures is evaluated [9]. Clinicians routinely diagnose steatosis by comparing the brightness of the liver and the kidney (Fig. 1). US images can be viewed immediately; therefore, US is a real‑time imaging technique that provides qualitative to semi‑quantitative assessment of structural information and enables detection of steatosis. US is widely available, inexpensive, quick, portable and free of ionising radiation, allowing repeated examinations. However, the technique is limited by operator dependency, shows low sensitivity for the detection of mild steatosis (<20–30% HL content) and, because of hampered penetration and increased dispersion of sound waves, exhibits reduced accuracy in individuals with obesity or in cases of deep hepatic tissue evaluation [10]. Transient elastography (TE) extends the principle of US by adding a mechanical vibrator that is capable of generating shear waves. Using US pulses to track the propagation speed of these shear waves through the tissue, the individual organ stiffness can be quantitatively assessed, which is why it is commonly used for fibrosis staging in MASLD (Fig. 2a) [11]. TE can also be used to screen for hepatic steatosis by assessing the controlled attenuation parameter (CAP), an algorithm based on total US attenuation at 3.5 MHz [12]. This makes TE a valuable dual‑purpose tool. Although TE shares the advantages of US, it requires a dedicated device and specific operator training. The accuracy of the method is also decreased at high BMI, similar to classical US.

### Computed tomography

**Fig. 3:**
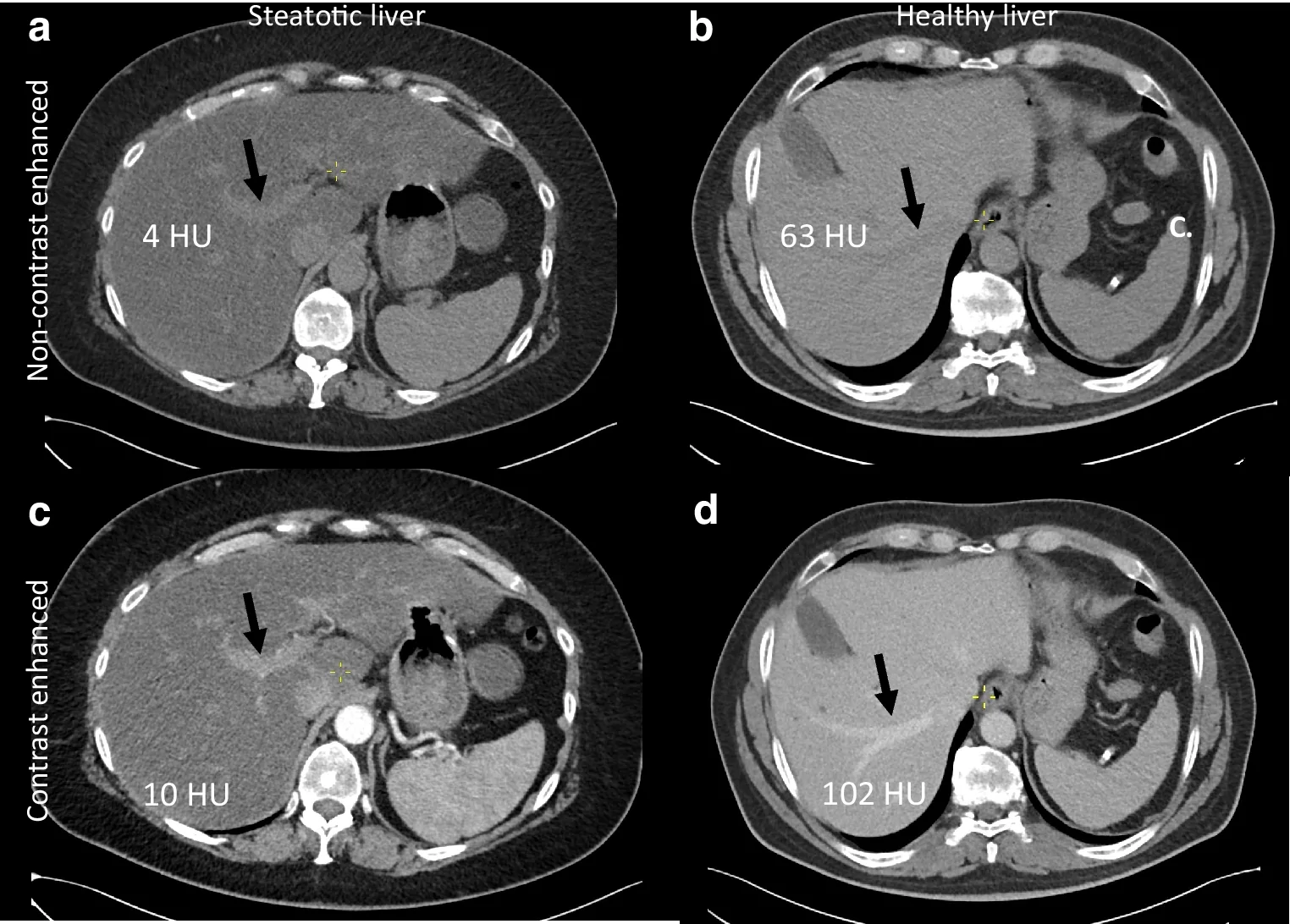
Representative CT images showing hepatic steatosis (**a**, **c**) and normal liver parenchyma (**b**, **d**) in two different individuals. (**a**) Non-contrast-enhanced CT of the upper abdomen showing a very low liver density (normal liver density 50–75 HU, steatosis if density is <40–48 HU). The hepatic veins and vena porta (arrow) are hyperdense compared with the liver. Even after contrast enhancement (**c**) there is only a slight increase in the HU value. (**b**) Non-contrast-enhanced normal liver parenchyma; the hepatic veins (arrow) are hypodense compared with the parenchyma. After contrast enhancement, lean liver shows a marked increase in contrast between vessels and parenchyma in the portal venous phase (**d**). Images courtesy of T. van de Weijer (Department of Radiology, Maastricht University Medical Center, Maastricht UMC+, the Netherlands). This figure is available as part of a downloadable slideset

Computed tomography (CT) is an x-ray-based technique that measures tissue-dependent attenuation of emitted ionising radiation to generate volumetric datasets of the organ of interest (Fig. 3). This technique provides high spatial resolution (submillimetre level) and can be used independently of body habitus, as contrast is provided by differences in tissue electron density. Volumetric reconstructions of the organ of interest are rapidly provided after a scan, making CT a qualitative and quantitative imaging modality for assessing hepatic morphology and density. CT is broadly available in hospital settings, relatively cost-effective for single examinations and fast, but its usage is limited by radiation exposure, which especially limits repeated assessments. It also shows low sensitivity for the detection of mild lipid accumulation, and hepatic iron overload can confound attenuation measurements and further reduce diagnostic accuracy for steatosis [13].

### MRI/magnetic resonance spectroscopy

**Fig. 4:**
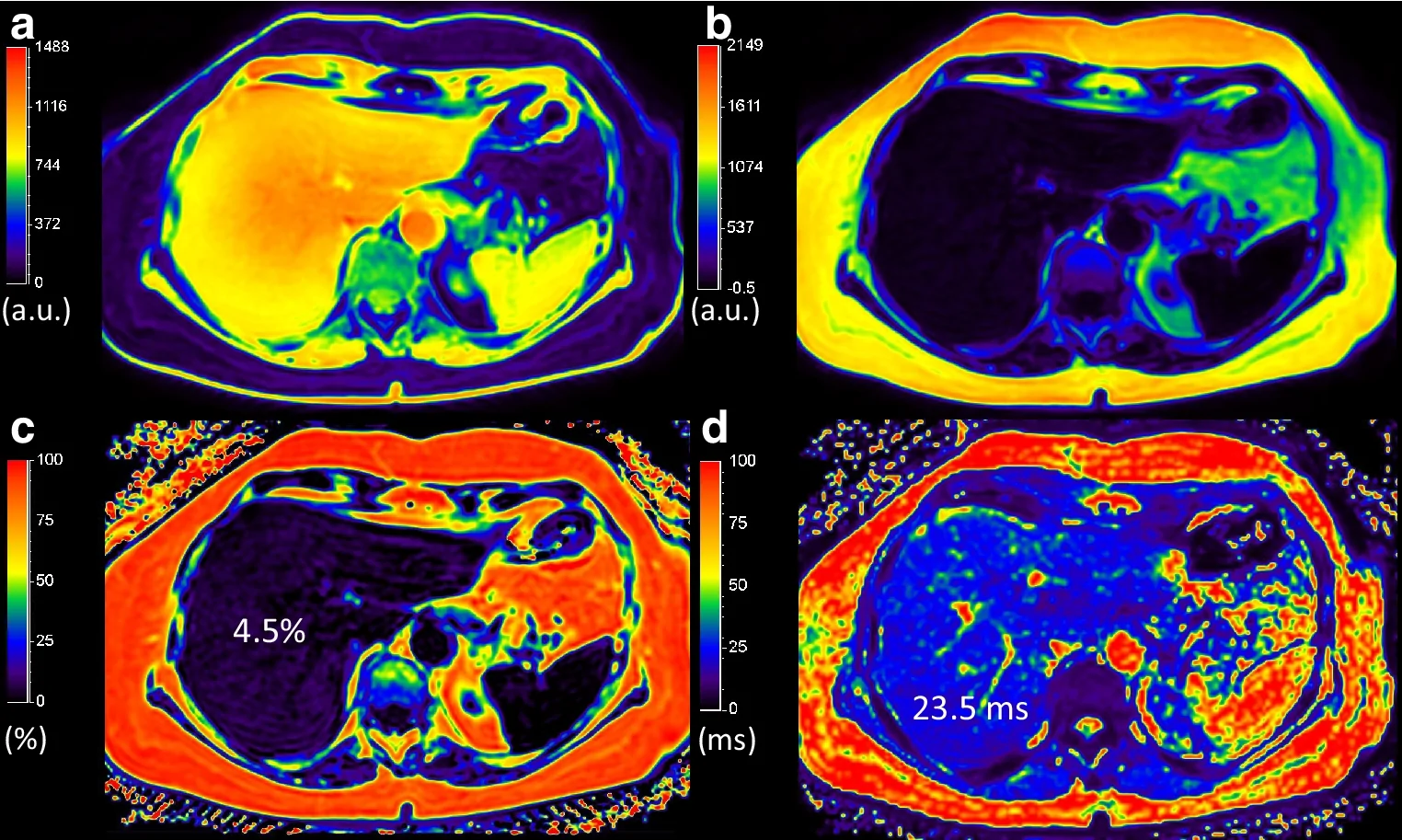
Exemplary results of an MRI-based Dixon sequence to assess the hepatic PDFF. Acquisition of a transverse multi-echo Dixon sequence is used to generate four outputs: (**a**) water-only image; (**b**) lipid-only image; (**c**) MRI-PDFF as lipid content (%); and (**d**) T_2_^*^ representing iron content (ms), which is used to correct the PDFF. The mean MRI-PDFF and T_2_^*^ are 4.5% and 23.5 ms, respectively. The images show the automatically processed output generated by the commercial vendor software. This figure is available as part of a downloadable slideset

The principle of MRI is based on the excitation and detection of nuclear spins (mainly protons, ^1^H) in the presence of a strong external magnetic field using radiofrequency pulses. The spatial resolution of MRI is high (millimetre level) and relatively independent of volunteer body habitus. The contrast in MRI is generated by differences in T_1_/T_2_ relaxation times and proton density of different tissues. An image is generated by spatial encoding during the scan, followed by image reconstruction after the scan, making it a powerful tool for qualitative and quantitative assessment of structural information. With MRI, the proton density fat fraction (PDFF), representing HL content, can be assessed using chemical shift-encoded gradient-echo MRI to obtain whole-liver fat fraction maps (Fig. 4) [14–16]. The PDFF is obtained from the ratio of the signal amplitude originating from triglyceride protons to the sum of the signal amplitude from triglyceride and water protons within each voxel. MRI is available in hospitals and specialised centres. Scans are expensive and require long examination times but are free of ionising radiation, making MRI an ideal tool for repetitive examinations. Contraindications to MRI include claustrophobia and certain (metal/electronic) implants. In addition, some sequences are prone to motion artefacts, and examination of individuals with severe obesity may be restricted by the size of the magnet bore.

Another technique, diffusion-weighted imaging (DWI) MRI, can be used to measure the mobility of water protons to access functional information based on tissue cellularity and microstructure [17, 18].

Magnetic resonance elastography (MRE) uses a vibration source to generate waves that propagate through the organ of interest, creating microscopic shear displacements that are imaged using a phase‑contrast MRI sequence [19]. The resulting 2D or 3D stiffness maps allow estimation of mean liver stiffness (MLS) over a large portion of the liver, and MRE is therefore often used for the assessment of fibrosis (Fig. 2b). However, the need for extra hardware, longer scan times and expertise limit its usage to specialised centres.

**Fig. 5:**
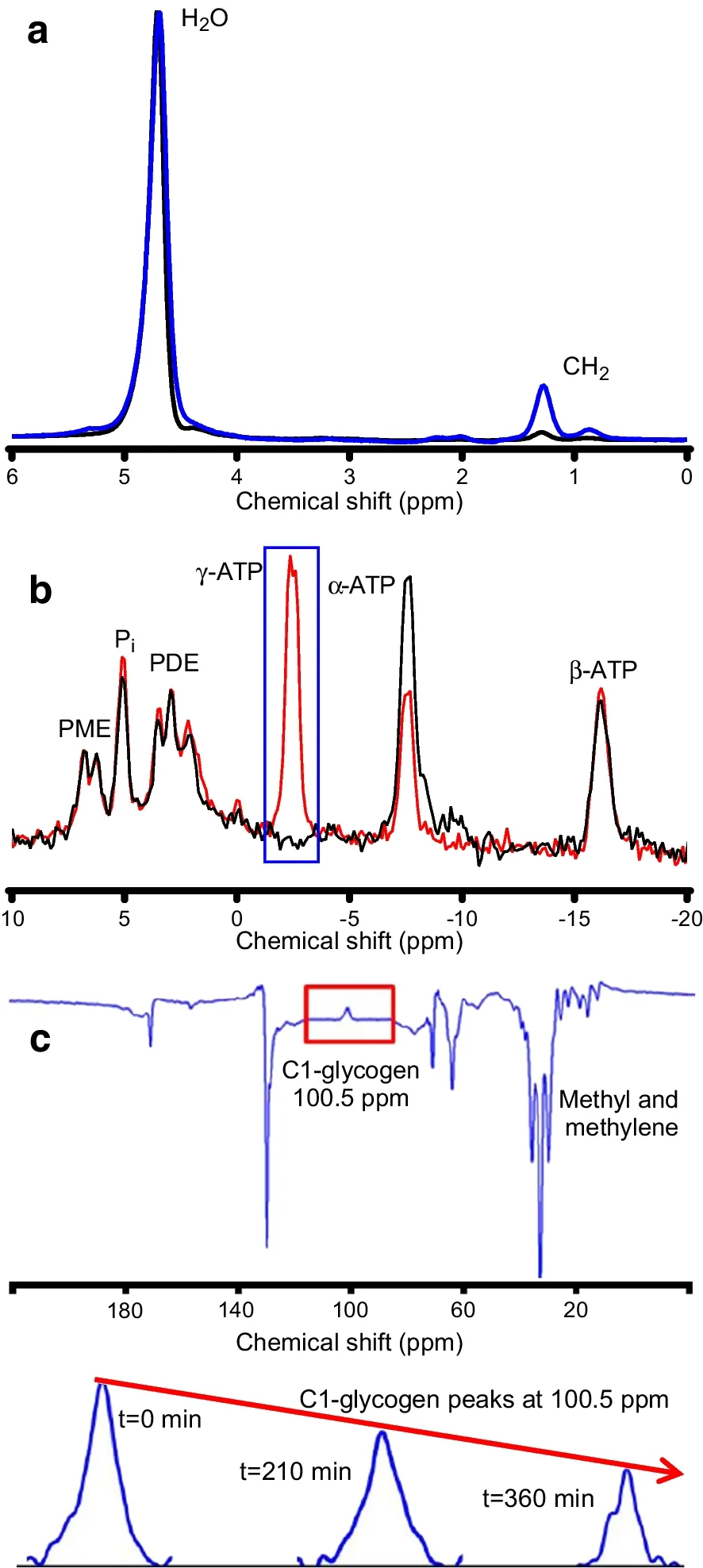
Representative MRS spectra from the human liver. (**a**) ^1^H-MRS to assess HL content. The spectra show results for two volunteers, with HL content of 9.2% (blue) and 1.5% (black). Peak intensities were normalised to the corresponding water signal. (**b**) ^31^P-MRS spectrum (red) for quantification of ^31^P-metabolites, and saturation transfer (ST) spectrum (black) for assessing ATP synthesis flux. The blue rectangle indicates the frequency at which the saturation pulse was applied (directly on γ-ATP), causing its signal to vanish; a decrease in inorganic phosphate (Pi) can be detected due to chemical exchange. (**c**) ^13^C-MRS spectrum for the detection of glycogen. Repetitive measurements allow glycogen dynamics to be assessed; a decrease in glycogen concentration in the fasting state is shown. Figure 5b adapted from [74] under the terms of the CC BY 4.0 Attribution License (http://creativecommons.org/licenses/by/4.0/). Figure 5c adapted from [84] under the terms of the CC BY 4.0 Attribution License (http://creativecommons.org/licenses/by/4.0/). This figure is available as part of a downloadable slideset

Magnetic resonance spectroscopy (MRS) extends MRI principles by allowing individual detection of frequency-specific signals from metabolites within a defined volume of interest, enabling metabolic profiling that is complementary to structural data obtained from MRI. HL content and composition can be assessed using proton (^1^H) MRS, while absolute quantification of high-energy metabolites such as ATP and inorganic phosphate (Pi), as well as carbohydrate metabolites such as glycogen, can be performed using phosphorus (^31^P) or carbon (^13^C) MRS (Fig. 5) [20, 21]. MRS is subject to the same availability and safety constraints as MRI, plus it requires additional expertise and hardware, longer examination times and even more (motion)-sensitive protocols.

### Positron emission tomography/single-photon emission computed tomography

Positron emission tomography (PET) and single-photon emission computed tomography (SPECT) are based on the detection of gamma photons emitted from injected radiotracers that reflect specific biological processes such as glucose metabolism (e.g. FDG-PET), perfusion, inflammation or fibrosis activity [22]. Their spatial resolution is moderate (typically 4–10 mm for PET, lower for SPECT) and largely independent of body habitus. Contrast is determined by tracer uptake and distribution. Image formation is based on tomographic reconstruction of coincidence photon events, making PET/SPECT powerful tools for functional and molecular assessment, complementing structural imaging. These modalities are available only in specialised nuclear centres, are expensive and time-consuming, and involve ionising radiation from tracers.

## Parameters of interest for the detection of MASLD

### Hepatic steatosis

Hepatic steatosis is defined as an HL content exceeding 5% of hepatocyte volume. This condition arises from impaired HL homeostasis, in which excessive NEFA influx from adipose tissue lipolysis and de novo lipogenesis exceeds β-oxidation and VLDL export. Imaging techniques have improved the diagnosis of MASLD by allowing direct measurement of HL content, which provides greater accuracy and sensitivity than blood tests [23].

US is able to detect moderate to severe steatosis (HL content >20–30%) through increased liver echogenicity (bright liver; see Fig. 1) and beam attenuation, with a pooled sensitivity of 84.8% and specificity of 93.6% (*n*=4720) compared with histology in a meta-analysis of diagnostic accuracy [24]. Furthermore, as outlined above, CT and special MRI and MRS sequences can also be used to assess steatosis, with MRI and MRS being considered the gold standards for non-invasive detection of HL content [25]. A population-based ^1^H-MRS study has suggested a HL content of around 5.6% as the upper limit of normal [26]. Histological assessment of steatosis remains semi-quantitative and is influenced by sampling variability and reader-dependent interpretation. Accordingly, histological steatosis grades should not be assumed to correspond directly to MR-based HL content, as comparative studies have shown that histology-derived values are typically higher, often by a factor of approximately 1.5–3 [27–29].

#### Diagnostic performance of US, CT and MRI/MRS

Comparing the two MR-based techniques MRI-PDFF and ^1^H-MRS, MRI-PDFF is typically faster, as it can be acquired in a single breath-hold, and has more widespread availability, whereas MRS requires additional expertise for spectral processing. In addition, major MRI vendors provide proprietary algorithms for automated inline PDFF map generation. Nevertheless, dedicated MRS protocols have also been developed that allow screening for steatosis within a single 15 s breath-hold [30]. A meta-analysis found that these MR methods have overall superior sensitivity and specificity for the evaluation of hepatic steatosis (MRI: 95% CI 82.0%, 97.4% and 76.1%, 95.3%; MRS: 95% CI 72.7%, 88.5% and 92.0%, 95.7%, respectively) compared with other techniques such as US (95% CI 73.3%, 90.5% and 69.6%, 85.2%, respectively) and CT (95% CI 46.1%, 72.0% and 88.1%, 94.6%, respectively) [31]. In a subgroup with an HL content <5%, CT especially showed a significantly lower mean sensitivity of 46.1% compared with 73.3% for US and >80% for MRI and MRS [31]. Another study examined the accuracy of MRI, CT and US for the quantification of hepatic steatosis with MRS as the reference standard (*n*=50 adults), reporting correlations of 0.98 for MRI-PDFF and 0.83 for CT [32]. When comparing MRS with US, a weighted kappa value of 0.82 was found when classifying steatosis into four grades. This study also confirmed the limited sensitivity of CT in individuals without steatosis, with a significantly lower correlation between MRS and CT of 0.04 [32].

#### Clinical practice

As CT examinations also involve ionising radiation exposure, US- and MR-based methods are the two dominant imaging modalities for the assessment of steatosis. While MRI/MRS provide unmatched quantitative precision, their high costs and limited availability restrict their use in low- and middle-income countries, where MASLD prevalence, often linked to rising rates of obesity and type 2 diabetes, is substantial (44.4% in Latin America and 36.5% in the Middle East and North Africa) [33]. Despite their qualitative nature and limited sensitivity for mild steatosis, US-based methods therefore remain the first-line screening modality recommended by both EASD and EASO because of their widespread availability, low cost, real-time results and suitability for resource-limited settings globally [2]. Recent advancements in US technology have enabled the provision of CAP, which combines HL content measurement with fibrosis assessment using TE (Fig. 2a). For identification of MASLD by CAP, an optimal threshold of 288 dB/m (95% CI 0.70, 0.90) was identified using MRI-PDFF as the reference [16]. However, a cross-sectional study showed that MRI-PDFF remains superior for steatosis detection compared with CAP [15]. A further development of this technique is the introduction of a US device that measures the US-guided attenuation parameter (UGAP) using B-mode US, allowing the liver to be imaged in real time and thereby enabling the operator to explicitly avoid regions with vessels or artefacts. A comparison between CAP and UGAP with MRI-PDFF as the gold standard reported superior correlation of UGAP in all stages of steatosis, with 100% feasibility of measurement not only in the supine position but also in the lateral position [34].

### Fibrosis and inflammation

Liver fibrosis is the strongest predictor of liver-related morbidity and mortality in MASLD [23], representing extracellular matrix (collagen) deposition by activated hepatic stellate cells in response to chronic injury, for example from lipid peroxidation and inflammation. Fibrosis progresses through stages F0–F4 (cirrhosis), with F ≥2 considered ‘clinically significant’ and F ≥3 considered ‘advanced’. MASH activity or inflammation encompasses lobular inflammation and hepatocyte ballooning superimposed on steatosis, driving fibrogenesis. Single conventional laboratory parameters such as ALT or FIB-4 have shown limited reliability for fibrosis assessment in MASLD/MASH, especially in individuals with type 2 diabetes, likely requiring subgroup-specific cut-offs because of higher disease prevalence. In a study in people with type 2 diabetes, the use of low ALT thresholds (>20 IU/l in women, >30 IU/l in men) would have identified 58% of participants as having MASH and 38% as having advanced fibrosis; FIB-4 demonstrated moderate standalone performance (area under the receiver operating characteristic curve [AUROC] 0.71 and 0.62, respectively [5]. A head-to-head comparison confirmed FIB-4’s inferior accuracy (AUROC 0.63 for fibrotic MASH) compared with imaging-based scores using TE or MRI, emphasising the need for adapted cut-offs [35]. Imaging modalities provide complementary assessment in this high-risk population.

#### US techniques

Non-invasive US-based elastography techniques such as TE have thus become first-line options because of their widespread availability, low cost and ability to simultaneously measure steatosis via CAP and fibrosis via liver stiffness measurement [11, 16, 24]. These methods are especially valuable in primary care and resource-limited settings, although accuracy can be affected by the presence of obesity [2, 36]. Conventional US is of limited use for the assessment of fibrosis and MASH activity, although some approaches have introduced US scoring systems for the prediction of MASH, such as the US fatty liver indicator (US-FLI). This indicator includes multiple sonographic parameters, for example liver/kidney contrast, the presence or absence of posterior attenuation of the US beam, vessel blurring, visualisation of the gallbladder wall and diaphragm, and areas of focal sparing [37].

#### Comparison of TE and MRE

The best non-invasive imaging marker of fibrosis is liver stiffness. After pressing on the liver mechanically, the propagation of the shear waves can be followed with either US (TE) or MRI (MRE) (see above). Although TE is the most widely used elastography technique for detecting fibrosis in MASLD, the technique has its drawbacks. As each TE acquisition samples only a small cylindrical region of liver parenchyma (10 × 40 mm) at a depth of 25–65 mm, multiple valid measurements at different locations are required, and care must be taken to avoid large vessels or biliary structures within the region of interest to prevent artefacts [38]. In individuals with obesity especially, TE is associated with a high rate of measurement failure or an unreliability rate of about 20% [11]. MRE, on the other hand, uses a conceptually similar shear wave-based technique for detecting liver stiffness as a marker of fibrosis and its resulting 2D or 3D stiffness maps allow estimation of MLS over a large portion of the liver, thereby reducing sampling error (Fig. 2b). Liver MRE is currently considered the most accurate non-invasive technique for the detection and staging of liver fibrosis [39]. Two meta analyses of studies comparing MRE with TE in terms of sensitivity and specificity reported that MRE is superior to TE in diagnosing each stage of liver fibrosis [40, 41]. However, it should be noted that both US TE and MRE are positively correlated with each other (*r*=0.88 in *n*=62) [42] and both modalities are also positively correlated with ballooning [14], which is not surprising, as the progression of MASLD often comprises both fibrosis and inflammation. Therefore, the MRE-MLS can also be used as a marker of disease progression and treatment response but, because of the high costs and limited availability of MRI combined with the need for MRE hardware and expertise, TE is more commonly applied in clinical practice.

#### Other MRI-based methods

Other approaches, such as DWI-MRI, may also be suitable for the detection of fibrosis. A recent study found that this technique may have the same diagnostic accuracy as MRE when combined with PDFF-based lipid correction in people with MASLD [17]. Another study reported that both the perfusion fraction and the pseudo-diffusion coefficient decrease in people with chronic liver disease, whereas MLS increases [18]. Moreover, this study found that the parameter tissue diffusivity can detect clinically significant portal hypertension, and it was suggested that combining diffusion MRI with MRE may enhance diagnostic accuracy. However, it should also be highlighted that MRI’s high spatial resolution and its ability to generate native and contrast-enhanced images enable texture analysis (TA) combined with machine learning (ML) for fibrosis classification using widely available sequences, thereby extending the use of MRI beyond hardware-dependent MRE. For example, a recent study demonstrated that using TA+ML on T_1_ relaxation maps achieved a performance comparable to that of MRE for distinguishing no to mild (F0–2) vs severe (F3 or F4) fibrosis (AUC 0.75 vs 0.76), whereas TA on T_2_ relaxation maps was outperformed by MRE (0.515 vs 0.759); combining MRE with TA on T_1_ relaxation maps further improved performance (AUC 0.82) [43]. Consistent with these findings, another study found that using TA+ML on non-contrast T_1_ images achieves comparable results to MRE (AUC 0.82 vs 0.92), while T_2_ fat-saturated images perform less well (AUC 0.57) [44].

Another MRI-based approach that has been suggested for the evaluation of fibrosis and inflammation is T_1_ mapping, which can differentiate between MASLD and MASH [38]. This technique measures the T_1_ relaxation time, which is related to the free water content, a factor known to be increased in inflammation and fibrosis [45]. As iron shortens the T_1_ relaxation time, and iron content in the liver can vary, T_1_ values need to be corrected for the influence of iron. A median iron-corrected T_1_ (termed cT_1_) of 745 ms was reported in individuals with a BMI ≥25 kg/m^2^ and PDFF >5%, compared with 666 ms in those with a BMI ≤25 kg/m^2^ and PDFF <5% [46]. Similarly, a literature review summarised that a significant decrease in cT_1_ of −79 ms, −68 ms and −62 ms was associated with treatment with fibroblast growth factor analogues, glucagon-like peptide-1 receptor agonists, and farnesoid X receptor agonist, respectively, over a median of 17 weeks, with the placebo groups showing no changes in cT_1_, making cT_1_ a sensitive marker for assessment of longitudinal treatment responses [47]. Whereas cT_1_ correlates with inflammation, ballooning and fibrosis, MRI-PDFF alone is only weakly associated with ballooning and is not linearly related to inflammation or fibrosis [48]. Importantly, however, the prolongation of T_1_ observed with fibrosis may also be partly due to increased lipid content. Therefore, T_1_ maps corrected for iron but not for lipid content may reflect both high free water content and/or high lipid content [49]. Therefore, information obtained from cT_1_ values is not specific for fibrosis alone. It has been reported that, when T_1_ values are corrected for lipid content, the correlation of cT_1_ relaxation time with fibrosis is weakened and loses statistical significance [48].

Another recently proposed method for fibrosis assessment is T_1_ρ mapping using continuous low-frequency and low-amplitude radiofrequency pulses. In preclinical MASLD models, hepatic T_1_ρ mapping provided superior diagnostic performance for the detection of histological fibrosis and portal hypertension compared with native T_1_ mapping and T_2_ mapping, probably because T_1_ρ mapping is less affected by steatosis and inflammation [50]. In a clinical study, this method showed an even higher correlation (*r*=0.75) with MRE than classical T_1_ mapping (*r*=0.49), indicating that T_1_ρ mapping is a more accurate marker of hepatic fibrosis in people with chronic and steatotic liver disease [51].

The use of dynamic contrast-enhanced (DCE)-MRI with a hepatocyte-specific contrast agent is usually not indicated in individuals with MASLD, except for advanced stages of liver disease (see ‘Liver function, tumours and cirrhosis’). However, interestingly, application of compartmental modelling to DCE-MRI data enables quantification of contrast agent uptake into and excretion from hepatocytes, and also provides estimates of the extracellular volume, a marker of fibrotic tissue [52]. Therefore, in principle, this may also provide a means for quantifying fibrosis.

#### PET-based methods

In many tissues, for example in the walls of blood vessels, FDG-PET is used as a non-specific marker of inflammation. In the liver, this is more complex, as glucose uptake and release are dynamically regulated depending on the metabolic state, without necessarily reflecting inflammatory processes. However, pilot data suggest that, when dynamic FDG-PET scanning is used and data are analysed using a multicompartment model, FDG transport from blood to hepatic tissue is significantly correlated (*r*=–0.73) with biopsy-based scores of inflammation and with the fatty liver disease activity score [53]. The addition of information on HL content from CT, which is acquired together with PET measurements, may improve the prediction of hepatic inflammation even further [54].

#### Future developments

Imaging developments in MASLD are increasingly focused on novel PET tracers and targeted MRI contrast agents to measure inflammation and fibrosis at the cellular level. For example, activated fibroblasts can be visualised using fibroblast activation protein (FAP) ligands and the uptake of the FAP tracer can be quantified. It has been shown that fibroblast activation protein inhibitor (FAPI) uptake correlates with alternative markers of fibrosis [55], such as the AST to Platelet Ratio Index (APRI), liver stiffness, and fibrosis grading based on biopsies [56]. Beyond FAPI, PET tracers that track resident and recruited macrophages have been developed and tested in murine MASH; these tracers may be helpful for monitoring MASH development in humans [57]. Activated hepatic stellate cells express platelet-derived growth factor receptor beta (PDGFRβ), and a corresponding PDGFRβ-targeted PET ligand has been developed [58]. In parallel, new MRI contrast agents are in development for more precise fibrosis detection. For example, liver-targeted iron oxide/dysprosium oxide nanoparticles show increased T_2_ relaxivity in murine models of liver fibrosis, enabling differentiation between early and moderate stages; these findings are consistent with biopsy-based fibrosis staging [59]. Similarly, hyaluronic acid-coated gadolinium-based nanoprobes selectively target hepatic stellate cells and exhibit high T_1_ relaxation rates, thereby enabling efficient MRI diagnosis of liver fibrosis [60]. More broadly, there is a growing body of review literature on nanotechnology-based probes for liver disease [61].

### Liver function, tumours and cirrhosis

MASLD increases the risk of HCC, particularly in advanced fibrosis/cirrhosis. Early HCC detection in cirrhotic livers is challenging owing to the presence of regenerative nodules and dysplastic lesions. Standard clinical imaging modalities for the detection of tumours are DCE-US, DCE-CT and DCE-MRI. DCE-US can quantify tissue perfusion based on phase-specific enhancement after the injection of microbubble contrast agents for treatment monitoring in oncology. However, its use is highly operator dependent, which is why new standardisation approaches have been recently reported [62]. In addition to standard DCE-MRI, which is very often applied in the clinic, liver-specific contrast agents are also available. When performing DCE-MRI with a hepatocyte-specific contrast agent (e.g. gadoxetate), a hepatobiliary phase of about 20 min is added to the dynamic phase for assessment of intracellular retention of the contrast agent [63]. Within this context, the functional liver imaging score (FLIS) was developed, calculated as the sum of three qualitative hepatobiliary phase features: parenchymal enhancement, biliary contrast excretion and portal vein signal intensity [64]. In a separate study, the quantitative parameters enhancement ratio at 15 min and contrast enhancement spleen index at 20 min were better predictors of morbidity and mortality in a cirrhotic cohort than commonly used clinical scores [65]. A recent study also incorporated parenchymal heterogeneity into FLIS and showed that this modified FLIS-H correlates significantly with fibrosis stages [66].

#### Beyond conventional imaging: ^31^P-MRS

A systematic review on the potential of ^31^P-MRS for assessing response to therapy in hepatopancreatobiliary cancer found that the phosphomonoester (PME)/ATP, PME/Pi and PME/phosphodiester (PDE) ratios were increased in people with cancer compared with control volunteers, and the PME/Pi ratio decreased consistently after therapy in all included studies [67]. In individuals with cirrhosis, an increase in PME/(PME+PDE) and decrease in glycerophosphorylethanolamine (GPC)/(PME+PDE) has been shown, indicating an accumulation of cell membrane precursors (PME) and a simultaneous decrease in cell membrane degradation products, indicating an overall decrease in membrane turnover [68].

### Energy and substrate metabolism

In contrast to the previous sections, which focus on morphological and structural changes such as steatosis and fibrosis, this section considers alterations in hepatic metabolism, which (at least in the early stages of MASLD) may be present without structural changes. Changes in metabolism, for example altered metabolite concentrations or fluxes, increase the risk for MASLD and may occur at a very early stage of disease, while other metabolic changes may be characteristic of disease progression. Examining metabolism, the key targets are intracellular energy status, mitochondrial function and substrate handling, which cannot be visualised by conventional anatomical imaging. These metabolic processes can only be probed non-invasively using spectroscopic and molecular techniques, specifically MRS and nuclear imaging methods such as PET and SPECT. Hepatic energy metabolism becomes dysregulated in MASLD and, especially on progression, is characterised by impaired ATP homeostasis, reduced mitochondrial oxidative phosphorylation capacity and altered glycogen storage dynamics [21, 69, 70].

#### ^31^P-MRS

Mitochondrial adaptations initially increase fatty acid oxidation but, in the longer term, mitochondrial function is hampered, culminating in bioenergetic failure characteristic of MASH [7, 8]. ^31^P-MRS is, in principle, capable of quantifying high-energy phosphates such as ATP, Pi, phosphocreatine (PCr), PME and PDE [71]. For example, studies investigating people with long-standing type 2 diabetes or people with overweight report significant decreases in both ATP and Pi concentrations, thought to reflect disturbed hepatic energy metabolism in the presence of obesity [72, 73]. As well as assessing absolute concentrations of ^31^P-metabolites, it is also possible to measure the unidirectional hepatic ATP synthesis rate using ^31^P-MRS saturation transfer, which is often regarded as a marker for mitochondrial function (Fig. 5b); however, exact interpretation is difficult, as glycolytic processes can also contribute to the observed ATP synthesis. Studies in type 1 and type 2 diabetes and in MASLD vs MASH have found an approximately 50% decrease in forward ATP synthesis rate, although the underlying causes may differ [74–76]. Some studies have also reported quantification of NADPH, with increased levels observed in MASH [68, 77]; however, at clinical field strengths, this resonance is usually not resolved from neighbouring signals such as NAD^+^ and α-ATP.

#### ^13^C-MRS

^13^C-MRS can provide insights into carbohydrate metabolism, especially by tracking hepatic glycogen synthesis/breakdown (natural abundance or on infusion of ^13^C1-glucose tracers) (Fig. 5c). By performing multiple measurements over time, net glycogenolysis during fasting or net glycogen synthesis after meal ingestion can be assessed [70, 78]. Insulin resistance, which is a common feature of MASLD, is known to hamper glycogen storage; however, presently, little is known about glycogen dynamics in MASLD. Hepatic oxidative metabolism can also be investigated using ^13^C-MRS. After infusion of ^13^C1-acetate, C1- and C5-glutamate are quantified to determine the mean rates of hepatic tricarboxylic acid (TCA) cycle and anaplerotic flux [79]. When the ^13^C-MRS-derived TCA flux is combined with ^31^P-saturation transfer measurements of the Pi to ATP flux, the efficiency of oxidative phosphorylation (ATP produced per unit TCA flux) can be assessed in vivo [80]. Other studies have performed hyperpolarised 1-^13^C pyruvate MRS for real-time quantification of both hepatic oxidative and glycolytic fluxes in vivo [81, 82].

#### Limitations of multinuclear MRS

Although multi-nuclei MRS is a very powerful tool for metabolic investigations, it is used mainly to help elucidate disease mechanisms in MASLD research, rather than for routine diagnostics. These examinations require additional hardware and specific expertise and, generally, involve long acquisition times.

#### Advanced PET techniques and tracers

Glucose uptake kinetics can be measured using PET in combination with FDG, the most commonly used radiotracer. The assessment of these parameters under physiological stimulation, for example during an OGTT or a hyperinsulinaemic–euglycaemic clamp, enables valuable data on insulin-mediated glucose uptake to be obtained [83]. However, additional information can be obtained by using more specialised tracers. A fluorinated fatty acid analogue, FHTHA, can be used to assess fatty acid uptake, while further detail can be obtained using other isotopes, such as ^11^C-acetate for assessing blood flow and oxidation or ^15^O-H_2_O for quantification of hepatic perfusion [22]. In principle, fat oxidative capacity can be assessed in vivo after injection of ^11^C acetate. While mechanistically informative, these techniques are very expensive and require highly specialised infrastructure, as the short-lived tracers necessitate on-site cyclotron facilities. This restricts their use to a few specialised centres and limits implementation in large population groups.

## Conclusion

Non-invasive imaging has transformed MASLD diagnostics, enabling a shift from dependence on biopsies to the use of multistep strategies combining blood scores, US, MRI-PDFF and elastography techniques (TE and MRE) as per EASL-EASD-EASO guidelines. Among these techniques, MRI-PDFF provides superior steatosis quantification, with a sensitivity and specificity of >75%. In addition, accuracy is high even when investigating individuals with <5% HL content, whereas US cannot reliably detect steatosis at very low HL levels. In fibrosis staging, MRE outperforms TE, although its routine use is limited by accessibility. Emerging techniques such as DWI-MRI, cT_1_-mapping, T_1_ρ-mapping, targeted PET tracers (FAPI, PDGFR) and ^31^P/^13^C-MRS (for metabolic insights: ATP, Pi, glycogen, ATP synthesis, TCA flux) enable comprehensive phenotyping for therapy monitoring amid increasing MASLD prevalence. The limitations of non-invasive imaging include operator dependency (US/TE), high costs (MRI/PET) and imaging artefacts associated with obesity. Future directions include the use of multiparametric scores, hepatocyte-specific agents and nanotechnology probes for cellular-level resolution. Ultimately, hybrid imaging and standardised protocols will enable precision medicine, improving early detection, progression tracking and personalised interventions in MASLD.

## Supplementary Information

Below is the link to the electronic supplementary material.

Slideset of figures (PPTX 3.97 MB)

## Data Availability

This article reviews only data extracted from published sources in the public domain.

## Abbreviations

ALT: Alanine aminotransferase
CAP: Controlled attenuation parameter
CT: Computed tomography
DCE: Dynamic contrast-enhanced
DWI: Diffusion-weighted imaging
FAPI: Fibroblast activation protein inhibitor
FIB-4: Fibrosis-4
HCC: Hepatocellular carcinoma
HL: Hepatic lipid
MASH: Metabolic dysfunction-associated steatohepatitis
MASLD: Metabolic dysfunction-associated steatotic liver disease
MLS: Mean liver stiffness
MRE: Magnetic resonance elastography
MRS: Magnetic resonance spectroscopy
PDFF: Proton density fat fraction
PDE: Phosphodiester
PET: Positron emission tomography
Pi: Inorganic phosphate
PME: Phosphomonoester
SPECT: Single-photon emission computed tomography
TCA: Tricarboxylic acid
TE: Transient elastography
US: Ultrasound
